# Distribution of Polymorphic and Non-Polymorphic Microsatellite Repeats in *Xenopus tropicalis*

**DOI:** 10.4137/bbi.s561

**Published:** 2008-02-26

**Authors:** Zhenkang Xu, Laura Gutierrez, Matthew Hitchens, Steve Scherer, Amy K. Sater, Dan E. Wells

**Affiliations:** 1 Department of Biology and Biochemistry, University of Houston, Houston, TX, U.S.A; 2 Human Genome Sequencing Center, Baylor College of Medicine, Houston, TX, U.S.A

**Keywords:** microsatellite, polymorphism, Xenopus genome

## Abstract

The results of our bioinformatics analysis have found over 91,000 di-, tri-, and tetranucleotide microsatellites in our survey of 25% of the *X. tropicalis* genome, suggesting there may be over 360,000 within the entire genome. Within the *X. tropicalis* genome, dinucleotide (78.7%) microsatellites vastly out numbered tri- and tetranucleotide microsatellites. Similarly, AT-rich repeats are overwhelmingly dominant. The four AT-only motifs (AT, AAT, AAAT, and AATT) account for 51,858 out of 91,304 microsatellites found. Individually, AT microsatellites were the most common repeat found, representing over half of all di-, tri-, and tetranucleotide microsatellites. This contrasts with data from other studies, which show that AC is the most frequent microsatellite in vertebrate genomes (Toth et al. 2000). In addition, we have determined the rate of polymorphism for 5,128 non-redundant microsatellites, embedded in unique sequences. Interestingly, this subgroup of microsatellites was determined to have significantly longer repeats than genomic microsatellites as a whole. In addition, microsatellite loci with tandem repeat lengths more than 30 bp exhibited a significantly higher degree of polymorphism than other loci. Pairwise comparisons show that tetranucleotide microsatellites have the highest polymorphic rates. In addition, AAT and ATC showed significant higher polymorphism than other trinucleotide microsatellites, while AGAT and AAAG were significantly more polymorphic than other tetranucleotide microsatellites.

## Introduction

Microsatellites are short tandem repeats of a DNA sequence that are highly abundant in the genomes of eukaryotes (Hearne et al. 1992; Tautz 1993; Schlotterer, 2000). The high levels of allelic variation, codominant inheritance, and ease of analysis have made these markers attractive for population genetics, genome mapping, pedigree studies, and forensic analyses (Wright and Bentzen, 1994; Ellegren, 2000). In spite of the promising aspects of microsatellites as useful molecular markers, little is known about their origin, evolution, organization, dynamics, and roles in genomes. Recently, with the exponential increase in the number of genomic sequences available for different organisms, bioinformatic approaches have been used to investigate the distribution and frequencies of different types of microsatellites (Toth et al. 2000; Katti et al. 2001; Subramanian et al. 2003; La Rota et al. 2005). Comparisons in the frequency and distribution of microsatellites among different eukaryotic genomes have revealed the most dominant microsatellite types vary across taxa (Toth et al. 2000).

*Xenopus laevis* and its diploid sister species *X. tropicalis* are among the major model systems for the fields of molecular, cell, and developmental biology. In the past several years, the genomic information on *Xenopus* has accumulated rapidly, and NCBI now carries over 1.25 million EST sequences for *X. tropicalis*. The Joint Genome Institute (JGI) has released the assembly version 4.1 of the *X. tropicalis* whole genome shotgun reads at a coverage of 7.65X (http://genome.jgi-psf.org/Xentr4/Xentr4.info.html). The present study represents part of our efforts to generate a genetic map for *X. tropicalis* using microsatellites as markers.

One of our initial steps in generation of the genetic map was to develop a large set of “nonredundant” microsatellite markers. In this context we define our nonredundant microsatellite markers as di-, tri-, and tetranucleotide microsatellites containing a minimum of five non-interrupted tandem repeats, which are embedded in single copy flanking sequences and thus (with proper primer design) can amplify a unique genomic location. The purpose of this manuscript is to investigate: (1) the distribution and frequency of perfect di-, tri-, and tetranucleotide microsatellites in the *X. tropicalis* genome; (2) the relative abundance of different repeat classes and motifs in nonredundant microsatellites; and (3) the variations in the rate of polymorphism within nonredundant microsatellites along with the factors, such as tandem repeat length and base composition, which affect these variations.

## Materials and Methods

### Animals

DNA samples from two unrelated *X. tropicalis* frogs from each of the two major inbred strains, Nigerian and Ivory Coast, were used for polymorphic analysis. Frogs and/or DNA samples were generously provided by R. Grainger, U. Va., and R. Harland, UC Berkeley. The JGI sequence data was obtained from a single female Nigerian frog.

### Estimation of frequencies of genomic microsatellites

*Xenopus tropicalis* genome assembly 4.1, generated by the Joint Genome Institute (JGI), Department of Energy (DOE) was used to estimate the distribution and frequencies of di-, tri-, and tetranucleotide microsatellites. For this study, all non-interrupted di-, tri-, and tetranucleotide microsatellites with 5 or more tandem repeats were analyzed. A total of 445 million bases, representing about 25% of the *Xenopus tropicalis* genome, was analyzed using a Perl script SSRIT (Temnykh et al. 2001). So as not to skew for microsatellites present only on long scaffolds, we analyzed 256 scaffolds ranging in size from 23,997 bp (Scaffold-2010) to 7,817,814 bp (Scaffold-1). The repeat motifs of di-, tri-, and tetranucleotide microsatellites were compressed into core groups in which different reading frames and complementary strand sequence were merged (Table 1). The results from output tables of SSRIT were analyzed using Microsoft Excel.

### Selection of non-redundant microsatellites and polymorphism testing

The term “nonredundant microsatellites” refers to di-, tri-, and tetranucleotide microsatellites containing a minimum of five non-interrupted tandem repeats that are embedded in single copy sequences. These microsatellites were identified by a bioinformatics screen from the *Xenopus tropicalis* genome assembly 2.0. The data mining script was based on the publicly available computer program, Tandem Repeats Finder (TRF) (Benson, 1999), and modified to find di-, tri-, and tetranucleotide microsatellites with more than 5 repeats embedded in unique flanking sequences suitable for primer design. Initially, nonredundant di-, tri-, and tetranucleotide microsatellites were identified randomly from the entire genome. Subsequently, identification of nonredundant microsatellites was targeted to underrepresented scaffolds. Once nonredundant tri- and tetranucleotide repeat sequences had been identified from all scaffolds that include them, the data mining script was further modified to identify primarily dinucleotide repeats.

Primer pairs with annealing temperature at 58 °C (± 2 °C) were designed and initially tested on agarose gels to confirm their amplification under standard conditions (58 °C, 1.5 mM Mg^+2^, and 30 cycles). All primer pairs that amplified single bands were tested for polymorphisms between Nigerian and Ivory Coast strains. Polymerase chain reaction conditions consisted of 10 ng DNA, 0.5 μM of forward and reverse primers, 1.5 mM MgCl_2_, 0.2 mM of dGTP, dCTP, dTTP, 0.02 mM of dATP, 0.05 U/μl of Taq, 1X buffer, and 0.07 μCi/ul of ^35^S dATP. PCR amplification profile is 94 °C for 4 min followed by 30 cycles of 94 °C for 1 min., 58 °C for 1 min and 72 °C for 2 min with a final elongation of 30 min at 72 °C. Amplified products were electrophoresed in polyacrylamide gels and visualized by autoradiography. The known sequence of the pGEM- 3zf(+) vector was used as a ladder to establish the size of the microsatellites.

### Statistical analyses

Significance of the differences in length of di-, tri-, and tetranucleotide microsatellites and the mean copy number of different motifs was determined by ANOVA. This step was followed by a post-test using the GraphPad Prism software, which employs the Bonferroni correction to adjust for multiple comparisons. Comparisons in average copy numbers between genomic and nonredundant microsatellites were carried out by Student’s t-tests. Contingency tables were used to compare the polymorphism among microsatellites with different lengths, different types of microsatellites, and different motifs.

## Results

### Distribution and frequencies of di-, tri-, and tetranucleotide microsatellites

A total of 91,304 perfect di-, tri-, and tetranucleotide microsatellites with a minimum of five tandem repeat units were identified in 444,970,789 bp (~ 25%) of the *X. tropicalis* genome (Table 2). The total length of perfect di-, tri-, and tetranucleotide sequence represented in this sample is 1,705,957 bp, representing 0.38% of the total DNA analyzed. Dinucleotide microsatellites account for 78.7% of identified microsatellites and significantly outnumber tri- and tetranucleotide microsatellites (p< 0.001). The average distance between two trinucleotide microsatellites (59.9 kb) is almost 10 times that of dinucleotide microsatellites (6.2 kb). Our analysis suggests that in every one million base pairs of genomic sequence, there are an average of 161 dinucleotide, 27 tetranucleotide, and 17 trinucleotide microsatellites.

**Figure 1:**
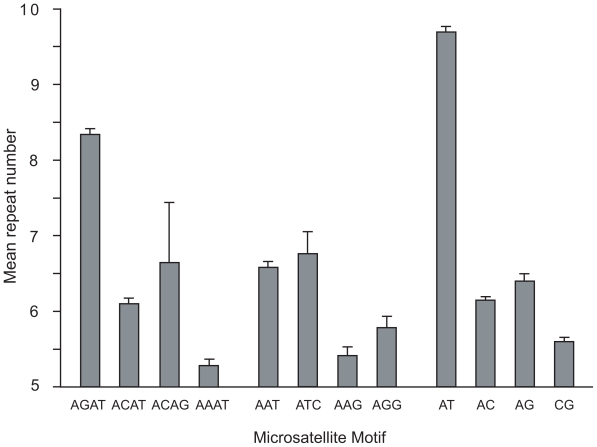
**Mean tandem repeat number of microsatellite motifs in genomic DNA.** Mean repeat numbers were determined for each di-, tri-, and tetranucleotide microsatellite containing a minimum of five perfect tandem repeats. Numbers for the entire genome were estimated from a survey of 444,970,789 base pairs (~25%) of the *X. tropicalis* genome. Only the four most prevalent motifs for each size class are shown. The AGAT tetranucleotide motif was significantly more common that other tetranucleotide motifs (p < 0.001). Similarly The AT dinucleotide motif was significantly more common that other dinucleotide motifs (p < 0.001). Standard errors are shown.

Among the di-, tri-, and tetranucleotide repeat classes of microsatellites, the most abundant repeat motifs are AT, AAT, and AGAT respectively (Table 2). These three repeat motifs account for more than 66% of the microsatellites present in the *X. tropicalis* genome, with the AT microsatellite alone representing over 50% of the total microsatellites in the genome. Figure 1 graphically shows the mean number of tandem repeats present in each of the four most abundant microsatellite motifs for each repeat class. Interestingly, for both the dinucleotide and tetranucleotide repeat classes, the most abundant motif also contained the highest number of tandem repeats, that is, both the AT and AGAT repeats were significantly longer than other di-, and tetranucleotide repeats (p < 0.001). However, this trend was not seen in the trinucleotide repeat class, as the ATC repeat class is not significantly more prevalent than the AAT repeat class.

### Relative abundance of nonredundant di-, tri-, and tetranucleotide microsatellites

**Figure 2:**
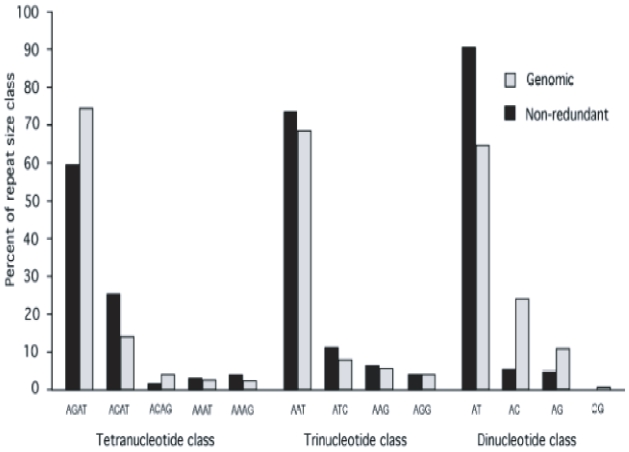
**Relative abundance in genomic and nonredundant DNA of each motif within each of the three microsatellite repeat size classes analyzed.** The abundance of each motif within both the genomic sample and the nonredundant sample is plotted against as a percentage of the abundance of the entire size class. AT, ATT, and AGAT were statistically more abundant than other members of their respective size class motif in both genomic and nonredundant samples. Only the most prevalent motifs for each size class are shown. Nonredundant results are shown in black and compared to genomic results are shown in gray.

As part of an ongoing effort to identify PCR amplifiable markers for use in developing a genetic map of *X. tropicalis*, data mining strategies were developed to identify microsatellites embedded in unique sequences suitable for unique genomic localization. To this end, we identified 5,128 non-redundant microsatellites, which were subsequently analyzed elsewhere for polymorphisms (see methods). The distribution and relative abundance of these nonredundant di-, tri-, and tetranucleotide microsatellites is shown in Table 3 and Figure 2. As was seen in the genomic survey, AT, AAT, and AGAT are also the most abundant nonredundant motifs, accounting for 90.30%, 73.52% and 59.48% of di-, tri-, and tetranucleotide motifs respectively (Table 3). Likewise, AC, ATC, and ACAT are the second most abundant motifs in their respective repeat classes. CG repeats, which were found in low numbers in the genomic survey, were absent from our set of nonredundant microsatellites.

**Figure 3:**
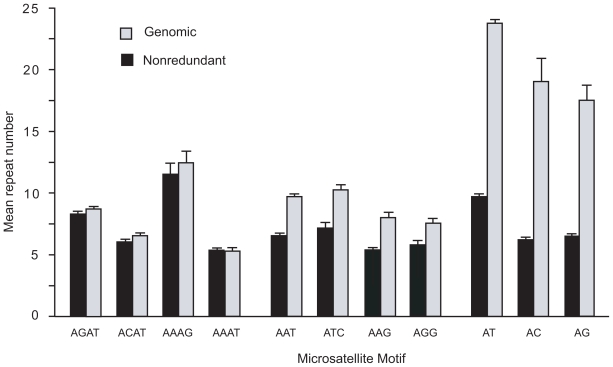
**Mean tandem repeat number in nonredundant DNA for each microsatellite motif.** Mean repeat numbers were determined for each di-, tri-, and tetranucleotide contained in our nonredundant microsatellite sample (see methods). Nonredundant results are shown in black, and genomic results are shown in gray. Only the most prevalent motifs for each size class are shown (no GC microsatellites were seen in our nonredundant sample). Standard errors are shown.

Table 4 shows a comparison in average number of repeat units between genomic and nonredundant di-, tri-, and tetranucleotide microsatellite repeat classes. In all cases, the nonredundant microsatellites have significantly longer repeats than their genomic counterparts (p < 0.001). This trend is also seen for most individual repeat motifs and is most pronounced for the dinucleotide motifs (Fig. 3).

### Polymorphism of di-, tri-, and tetranucleotide microsatellites

#### Effects of repeat length on the degree of polymorphism within microsatellites

**Figure 4:**
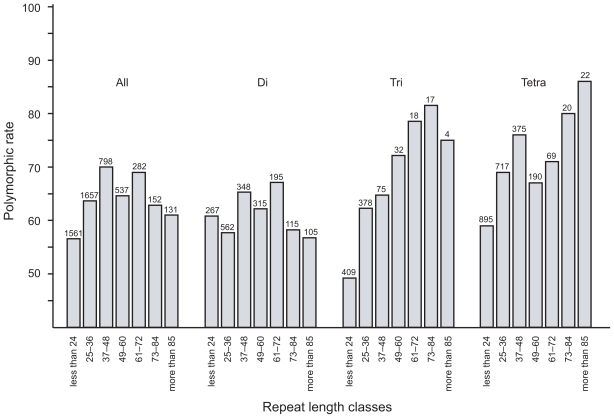
**Polymorphism rate for repeat length classes of each nonredundant microsatellite motif.** Each microsatellite motif was subdivided into seven groups based on the length of their core repeat sequences. The total number of loci analyzed is shown in each length class.

To examine the relationship between repeat length and degree of polymorphism, microsatellite loci were classified into seven groups based on the length of their core repeat sequences. The percent of each group that is polymorphic is displayed graphically in Figure 4 for each repeat length group. Clear trends can be observed for the tri- and tetranucleotide microsatellites showing a correlation between repeat length and degree of polymorphism. To determine if these trends were statistically significant, each microsatellite motif was divided into two length classes. Loci with a motif length 30 bp or less were designated as Class I markers, while those more than 30 bp were designated as Class II markers. Analysis of these groups revealed the Class II markers exhibited a significantly higher degree of polymorphism than Class I markers for all the three microsatellite repeat classes (di-, tri-, and tetranucleotide) (Table 5). This strongly suggests that repeat length does affect the degree of polymorphism for microsatellites.

#### Variations in polymorphism among different types of microsatellites

Statistical analysis further indicates the polymorphic rates of the three repeat classes of microsatellites analyzed are significantly different (Table 5). Here, “polymorphism rate” refers to the proportion of microsatellites in a given class that were shown to be polymorphic among individuals from the two strains of *X. tropicalis*. The pairwise comparisons show that tetranucleotide microsatellites have the highest polymorphic rates, significantly higher than dinucleotide and trinucleotide microsatellites (p < 0.01). Specifically within the Class II markers, tetranucleotide microsatellites also exhibit the highest rate of polymorphism; however, this difference is significant only for dinucleotide microsatellites (p < 0.01), and not for trinucleotide microsatellites. In Class I markers tetranucleotide microsatellites also exhibit a significantly higher polymorphism rate than trinucleotide loci (p < 0.05). However the polymorphism rate for tetranucleotide microsatellites was not seen to be significantly higher than dinucleotide microsatellites (p = 0.21).

#### Variations in polymorphism among different motifs of microsatellites

**Figure 5:**
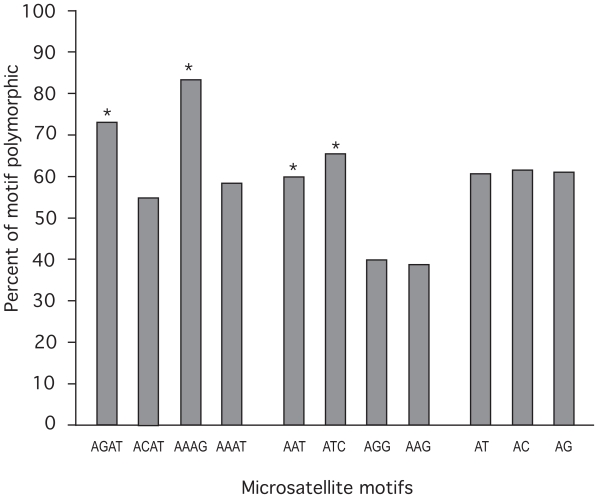
**Comparison of polymorphic rates among different microsatellite motifs.** Nonredundant microsatellites were tested for polymorphism as described in the methods section. *AAT and ATC show significant higher polymorphism than AAG and AGG (p < 0.001). *AGAT and AAAG are significantly more polymorphic than ACAT and AAAT (p < 0.001).

Figure 5 shows the rate of polymorphism for the most common microsatellite motifs. Although there was no significant difference in the rate of polymorphism among the three dinucleotide motifs (AT, AC, and AG), among the four most abundant trinucleotide motifs, AAT and ATC show significantly higher polymorphism than AAG and AGG (p < 0.01). Likewise, the most abundant tetranucleotide motifs, AGAT and AAAG, are significantly more polymorphic than ACAT and AAAT (p < 0.01). The higher polymorphism of microsatellites with motifs of AAT, ATC, AGAT, and AAAG seem to be correlated with their relatively longer repeat length (Fig. 3).

## Discussion

### Characteristics of X. tropicalis genome and the distribution of microsatellites

Our bioinformatics analysis found over 91,000 di-, tri-, and tetranucleotide microsatellites in ~25% of the *X. tropicalis* genome, suggesting there may be over 360,000 within the entire genome. Within the *X. tropicalis* genome, dinucleotide (78.7%) microsatellites vastly out-number tri- (8.1%) and tetranucleotide (13.2%) microsatellites. Although, there is some variation in the literature, these observations generally agree with data from other vertebrates (Toth et al. 2000). In the present study, the trinucleotide repeats are the least abundant of the microsatellites, which is consistent with studies in other vertebrates as well. Trinucleotide repeats, however, are more prevalent in protein coding regions, while di- and tetranucleotide repeats are scarce in exons (Li et al. 2002, 2004; Morgante et al. 2002; Toth et al. 2000; Dieringer and Schlotterer, 2003). The latter is probably the result of negative selection against frameshift mutations, which limits the expansion of microsatellites in coding sequences (Metzgar et al. 2000). During our analysis of the three types of microsatellites in scaffolds from the *Xenopus tropicalis* genome assembly 4.1, we noticed trinucleotide repeats were over-represented in some scaffolds and underrepresented in others. This could enable us to distinguish exon-rich scaffolds from those scaffolds containing primarily intergenic regions.

In the *X. tropicalis* genome the AT-rich repeats are overwhelmingly dominant. All three most abundant motifs in the three types of microsatellites (AT, AAT, and AGAT) are AT-rich (Table 2). Among all the di-, tri-, and tetranucleotide repeats identified, 51858 out of 91304 repeats (56.8%) are 100% AT repeats (e.g. AT, AAT, AAAT, and AATT), while 90128 (99%) repeats have an AT content not less than 50%. The high abundance of the AT-rich repeats in *X. tropicalis* could be partly attributable to the low melting temperature of AT-rich fragments and high mutation rates in poly (A/T) tracts (Prasad et al. 2005). However, these factors cannot explain why different taxa have different abundant repeat motifs.

Although exceptions exist (Schug et al. 1998), AC repeats have been reported as the most common dinucleotide repeats in most animals, including humans (Beckmann and Weber, 1992; Nadir et al. 1996; Katti, 2001), primates (Jurka and Pethiyagoda, 1995; Toth et al. 2000), rodents (Beckmann and Weber, 1992; Toth et al. 2000), chickens (Moran, 1993), *Fugu* (Edwards et al. 1998), bivalves (Cruz et al. 2005), and *Drosophila* (Schug et al. 1998; Bachtrog et al. 1999). In contrast, AG repeats are found to be the most abundant dinucleotide repeats in honey bees (Estoup et al. 1993) and yellowjacket wasps (Thoren et al. 1995), while AT repeats dominate the dinucleotide microsatellites in silkworms (Prasad et al. 2005) and yeast (Toth et al. 2000). Significantly, the predominance of AT repeats in the *X. tropicalis* genome found in the present study is the first such report in vertebrates. Interestingly, our results differ from those of Toth et al. (2000) who found that AC repeats are the most abundant repeats in vertebrates, occurring more than twice as frequently as AT repeats. In their study, 12.15% of the vertebrate taxonomic group was represented by *Xenopus laevis,* sister species of *X. tropicalis*. Further analysis is needed to determine whether the distribution of repeat motifs observed in *X. tropicalis* is characteristic of *Xenopus laevis* or other closely related frog species.

In contrast with dinucleotide abundance levels, the most prevalent tri- and tetranucleotide repeats of *X. tropicalis,* AAT and AGAT, are consistent with the results in some other vertebrates including *X. laevis*, although differing from those seen in some mammalian species (Edwards et al. 1998; Toth et al. 2000).

Schlotterer (2000) has suggested that taxon-specific predominance of different repeat motifs could be influenced by a different base composition in the genome as well as differences in the DNA mismatch repair systems. In addition, Prasad et al. (2005) have suggested there is a potential relationship between distribution of repeat motifs and higher-order chromatin structure. Tetranucleotide microsatellites containing the AGAT (GATA) motif are known to be associated with the sex chromosome in humans and to play a role in higher order chromatin organization and function (Singh et al. 1994; Zhao et al. 1995; Subramanian et al. 2003). *X. tropicalis* certainly provides a unique opportunity for comparative studies on the role of AGAT repeats because of the predominance of AGAT repeats in its genome.

### Comparisons between nonredundant and genomic microsatellites

The nonredundant di-, tri-, and tetranucleotide microsatellites, which were used as candidate markers for our linkage map, were independently identified from the *X. tropicalis* genome. Criteria for identifying nonredundant markers are that they have unique flanking sequences, that are long enough and have sufficient complexity to enable the design of unique PCR primers (Sharrocks, 1994). Among the three repeat types of nonredundant microsatellites analyzed, the distribution pattern of different motifs is generally consistent with that of genomic repeats, in that the most abundant di-, tri-, and tetranucleotide nonredundant motifs are AT, AAT, and AGAT. Although, this subset of microsatellite loci is similar to those identified in the entire *X. tropicalis* genome, the relative abundance of different motifs within di-, tri-, or tetranucleotide microsatellites show some divergence between nonredundant versus genomic repeats. For example, the AT repeats account for 64.7% of the total dinucleotide genomic loci, but 90.3% of the nonredundant dinucleotide loci respectively, suggesting a smaller proportion of AC and AG repeats are embedded in unique sequences with long enough flanking sequences for useful primer design. The discrepancies between the abundance of specific motifs in non-redundant loci versus genomic microsatellites may result from the appearance of AC or AG repeat strings embedded within more complex repetitive sequences. Large complex minisatellite repeats comprise over 1% of the *X. tropicalis* genome, with our initial surveys suggesting sequences containing AC repeats appear in very high copy numbers in these minisatellites. Inclusion of AC or AG repeat strings in larger, more complex minisatellite sequences could skew the distribution of repeat motifs among genomic microsatellites.

### Factors affecting microsatellite variation

It is well known that microsatellites are hot spots for genome mutation and variation (Weber, 1990; Ellegren, 2004). The variability seen in microsatellites is primarily due to sequence length polymorphisms caused by variable numbers of tandem repeats (Ellegren, 2000, 2004; Neff and Gross, 2001). In the present study, we compared the percentage of polymorphic loci in two different size classes (class I: length ≤ 30 bp; class II: length >30 bp). We found class II markers are significantly more polymorphic than class I markers for all three microsatellite repeat types. This suggests loci with larger numbers of repeats are more prone to mutation/expansion than those with fewer repeats. This result is consistent with other observations based on pedigree analyses (Brinkmann et al. 1998; Schug et al. 1998; Bachtrog et al. 2000; Kayser et al. 2000) and population genetics studies (Goldstein and Clark, 1995). The correlation between repeats length and the variability of microsatellites is understandable according to the replication slippage model, which is one of the widely accepted mutation mechanisms (Levinson and Gutman, 1987), as the longer the repeats, the more chances exist for the slipped-strand mispairing to occur.

Repeat type is another factor that has been found to affect stability of microsatellites (Schlotterer, 2000; Ellegren, 2004). Our study compared the polymorphism rate of 1,907 di-, 933 tri-, and 2,288 tetranucleotide microsatellites. The results indicate the tetranucleotide microsatellites have the highest rate of polymorphism while the dinucleotide microsatellites are the least polymorphic. Our results agree with Weber and Wong’s observation (1993) that the mutation rate for tetranucleotides is almost four times higher than that of dinucleotide repeats. Sia et al. (1997) reported similar mutation rates for tetranucleotide and dinucleotides repeats. However, two subsequent studies using different methodologies (Chakraborty et al. 1997; Lee et al. 1999) reached the conflicting conclusion that dinucleotide microsatellites have higher mutation rates than tetranucleotide microsatellites. However, the discrepancies between these studies may have resulted from insufficient data. It is worth noting that all three studies used only a small number of loci: Weber and Wong used 19 loci, Sia et al. used one di- and one tetranucleotide loci, Chakraborty et al. used 30 loci, and Lee et al. used two loci. Additional analysis is required to clarify the effects of repeat type on the polymorphism of microsatellites.

It has also been reported that the base composition of the repeat motifs may play a role in the variations of microsatellites. When they compared slippage rates between different microsatellites with different base compositions in *Drosophila* using an *in vitro* replication system, Schlotterer and Tautz (1992) found that sequences with high AT content mutate faster than those with high GC content. In contrast, Bachtrog et al. (2000) found that GT/CA-containing microsatellites of *D. melanogaster* had the highest mutation rate, while the AT-containing microsatellites had the lowest. Still another study showed that the CA and GA repeats of similar length in *Escherichia coli* genome exhibit similar mutability (Eckert and Yan, 2000). Although, our results indicate there are no differences in the polymorphic rate among the three dinucleotide motifs (AT, AC, and AG), among those most predominant tri- and tetranucleotide microsatellites, AAT and ATC exhibit a higher rate of polymorphism than AAG and AGG, and AGAT and AAAG are more frequently polymorphic than ACAT and AAAT. It remains unclear if the higher variability in AAT, ATC, AGAT, and AAAG microsatellites is a universal or species-specific phenomenon. In humans, an AAAG tetranucleotide locus has also demonstrated hypermutability (Talbot et al. 1995). It is worth noting that of the four tri- and tetranucleotide microsatellite motifs showing the highest rate of polymorphism, all have a higher number of repeat units per loci than their less polymorphic members. However, this trend does not hold for the dinucleotide loci as AT loci have significantly more tandem repeats than either AC or AG loci, yet the rate of polymorphism of AT does not significantly differ from the other two.

## Figures and Tables

**Table 1:** Core groupings of microsatellite motifs.

Dinucleotides	Trinucleotides	Tetranucleotides
**AC** (CA, GT, TG)	**AAC** (CAA, ACA, TTG, TGT, GTT)	**AAAC** (AACA, ACAA, CAAA, TTTG, TTGT, TGTT, GTTT)
**AG** (GA. CT, TC)	**AAG** (AGA, GAA, CTT, TTC, TCT)	**AAAG** (AAGA, AGAA, GAAA, TTTC, TTCT, TCTT, CTTT)
**AT** (TA)	**AAT** (ATA, TAA, ATT, TTA, TAT)	**AAAT** (ATAA, AATA, TAAA, TTTA, TTAT, TATT, ATTT)
**CG** (GC)	**ACC** (CAC, CCA, TGG, GGT, GTG)	**AACC** (CAAC, CCAA, ACCA, TTGG, GTTG, GGTT, TGGT)
	**ACG** (TCG, CGT, GAC, GTC, CGA)	**AACG** (GAAC, CGAA, ACGA, TTCG, TCGT, CGTT, GTTC)
	**ACT** (CTA, TAC, AGT, GTA, TAG)	**AACT** (ACTA, CTAA, TAAC, AGTT, GTTA, TTAG, TAGT)
	**AGC** (GCA, CAG, GCT, CTG, TGC)	**AAGC** (CTTG, TTGC, TGCT, GCTT, AGCA, GCAA, CAAG)
	**AGG** (GGA, GAG, CCT, CTC, TCC)	**AAGG** (AGGA, GGAA, GAAG, CCTT, CTTC, TTCC, TCCT)
	**ATC** (TCA, CAT, GAT, ATG, TGA)	**AAGT** (ACTT, CTTA, TTAC, TACT, TAAG, GTAA, AGTA)
	**CCG** (GCG, CGG, GCC, GGC, CGC)	**AATC** (TCAA, CAAT, ATCA, TTGA, TGAT, GATT, ATTG)
		**AATG** (ATGA, TGAA, GAAT, CATT, ATTC, TTCA, TCAT)
		**AATT** (ATTA, TTAA, TAAT)
		**ACAG** (CAGA, AGAC, GACA, CTGT, TGTC, GTCT, TCTG)
		**ACAT** (CATA, ATAC, TACA, ATGT, TGTA, GTAT, TATG)
		**ACCC** (CCCA, CACC, CCAC, GGTG, GGGT, TGGG, GTGG)
		**ACCG** (CGAC, GACC, CCGA, TCGG, CGGT, GGTC, GTCG)
		**ACCT** (GGTA, GTAG, TAGG, AGGT, CCTA, CTAC, TACC)
		**ACGC** (GCAC, CACG, CGCA, TGCG, GCGT, CGTG, GTGC)
		**ACGG** (CGGA, GGAC, GACG, CCGT, CGTC, GTCC, TCCG)
		**ACGT** (CGTA, GTAC, TACG)
		**ACTC** (CTCA, TCAC, CACT, GAGT, AGTG, GTGA, TGAG)
		**ACTG** (CTGA, TGAC, GACT)
		**AGAT** (GATA, ATAG, TAGA, ATCT, TCTA, CTAT, TATC)
		**AGCT** (GCTA, CTAG, TAGC)
		**AGGC** (GGCA, GCAG, CAGG, GCCT, CCTG, CTGC, TGCC)
		**AGGG** (GGGA, GGAG, GAGG, CCCT, CCTC, CTCC, TCCC)
		**ATCC** (CATC, TCCA, CCAT, GATG, ATGG, TGGA, GGAT)
		**ATCG** (GATC, TCGA, CGAT)

**Table 2:** Distribution of microsatellites in 25% of the *X. tropicalis* genome.

Repeat type	Motif	Number of loci	% of total loci	% of repeat type loci	Number of loci/Mbp	Loci Interval distance Kbp
Di-	AT	46488	50.92	64.72	104.47	9.57
	AC	17221	18.86	23.98	38.70	25.84
	AG	7851	8.60	10.93	17.64	56.68
	CG	267	0.29	0.37	0.60	1666.56
	Total	71827	78.67	100.00	161.42	6.20
Tri-	AAT	5080	5.56	68.35	11.42	87.59
	ATC	580	0.64	7.80	1.30	767.19
	AAG	409	0.45	5.50	0.92	1087.95
	AGC	344	0.38	4.63	0.77	1293.52
	AGG	292	0.32	3.93	0.66	1523.87
	AAC	272	0.30	3.66	0.61	1635.92
	ACT	245	0.27	3.30	0.55	1816.21
	ACC	102	0.11	1.37	0.23	4362.46
	ACG	59	0.06	0.79	0.13	7541.88
	CCG	49	0.05	0.66	0.11	9081.04
	Total	7432	8.14	100.00	16.70	59.87
Tetra-	AGAT	8973	9.83	74.50	20.17	49.59
	ACAT	1677	1.84	13.92	3.77	265.34
	ACAG	441	0.48	3.41	0.99	1009.00
	AAAT	272	0.30	2.26	0.61	1635.92
	AAAG	255	0.28	2.12	0.57	1744.98
	AAGG	90	0.10	0.75	0.20	4944.12
	AAAC	64	0.07	0.53	0.14	6952.67
	AACT	31	0.03	0.26	0.07	14353.90
	AGGC	29	0.03	0.24	0.07	15343.82
	AGGG	27	0.03	0.22	0.06	16480.40
	AATC	26	0.03	0.22	0.06	17114.26
	AATG	26	0.03	0.22	0.06	17114.26
	AATT	18	0.02	0.15	0.04	24720.60
	AAGT	16	0.02	0.13	0.04	27810.67
	ATCC	16	0.02	0.13	0.04	27810.67
	ACGT	15	0.02	0.12	0.03	29664.72
	ACTG	14	0.02	0.12	0.03	31783.63
	ACTC	13	0.01	0.11	0.03	34228.52
	ACCT	12	0.01	0.10	0.03	37080.90
	AACC	8	0.01	0.07	0.02	55621.35
	AACG	7	0.01	0.06	0.02	63567.26
	ACCC	5	0.01	0.04	0.01	88994.16
	AAGC	4	0.00	0.03	0.01	111242.70
	AGCT	3	0.00	0.02	0.01	148323.60
	ACGC	2	0.00	0.02	0.00	222485.39
	ATCG	1	0.00	0.01	0.00	444970.79
	Total	12045	13.19	100.00	27.07	36.94
Total		91304	100		205.1914	4.8735

**Table 3:** Distribution of nonredundant microsatellites in *X. tropicalis.*

Repeat Type	Motifs	Number	Abundance (% of repeat type)
Di-	AT	1722	90.30
	AC	100	5.24
	AG	85	4.46
	Total	1907	100
Tri-	AAT	686	73.52
	ATC	104	11.15
	AAG	53	5.68
	AGG	38	4.07
	ACT	27	2.89
	AGC	11	1.18
	AAC	6	0.64
	ACG	5	0.54
	ACC	3	0.32
	Total	933	100
Tetra-	AGAT	1361	59.48
	ACAT	603	26.35
	AAAG	86	3.76
	AAAT	62	2.71
	ACAG	36	1.57
	AAAC	16	0.70
	AAGG	14	0.61
	AACT	13	0.57
	ACGT	12	0.52
	AGGC	11	0.48
	AATT	10	0.44
	AATG	10	0.44
	ACCT	10	0.44
	AAGT	9	0.39
	ACTC	8	0.35
	AATC	6	0.26
	AACG	5	0.22
	ATCC	4	0.17
	AACC	4	0.17
	AGGG	3	0.13
	ACGC	2	0.09
	AAGC	1	0.04
	ACTG	1	0.04
	ACGG	1	0.04
	Total	2288	100
Total		5128	

**Table 4:** Comparison in mean repeat size between genomic and nonredundant microsatellites.

	# of repeat units		# of repeat units	

	Genomic	S.E.	Nonredundant	S.E.
Di-	8.33	0.27	23.96*	0.06
Tri-	6.28	0.16	9.58*	0.07
Tetra-	7.43	0.07	7.99*	0.07

* The nonredundant microsatellites have significantly longer repeats than their counterparts (student t-tests: for dinucleotides t = 57.02, df = 2958, p < 0.001, for trinucleotides t = 18.80, df = 1686, p < 0.001, for tetranucleotides t = 5.67, df = 4941, p < 0.001).

**Table 5:** Comparison of polymorphic and non-polymorphic markers by repeat size.

		Class 1 (≤30 bp)	Class 2 (>30 bp)	Total
Di*	Not Polymorphic	254 (43.1%)	496 (37.66%)	750 (39.33%)
	Polymorphic	336 (56.9%)	821 (62.34%)	1157 (60.67%)
	Total	590	1317	1907
Tri**	Not Polymorphic	324 (45.8%)	70 (31.0%)	394 (42.2%)
	Polymorphic	383 (54.2%)	156 (69.0%)	539 (57.8%)
	Total	707	226	933
Tetra**	Not Polymorphic	481 (39.8%)	290 (26.90%)	873 (35.09%)
	Polymorphic	728 (60.2%)	789 (73.10%)	1615 (64.91%)
	Total	1209	1079	2288
All**	Not Polymorphic	1191 (43.11%)	856 (32.66%)	2047 (38.02%)
	Polymorphic	1572 (56.89%)	1765 (67.34%)	3337 (61.98%)
	Total	2506	2622	5128
		+	++	++

Comparing polymorphism rates between Class 1 and 2 for each microsatellite repeat group (di, tri-, and tetra-),

* means the 2 size classes are significantly different (p < 0.05),

** means highly significantly different (p < 0.01). Comparing polymorphism rates among the three microsatellite repeat groups (di, tri-, and tetra-), + means significant differences (p < 0.05), ++ means highly significantly differences (p < 0.01) (see text).
